# Bidirectional network hubs: NT-genes as candidate targets for partial cancer reversal

**DOI:** 10.3389/fsysb.2026.1868020

**Published:** 2026-09-08

**Authors:** Gabriel Gil, Rolando Perez, Augusto Gonzalez

**Affiliations:** 1 Institute of Cybernetics, Mathematics and Physics, Havana, Cuba; 2 Center for Molecular Immunology, Havana, Cuba

**Keywords:** combination therapy, gene deregulation networks, NT-genes, phenotype reversal, tumor escape

## Abstract

**Background:**

Reversing the tumor phenotype is a long-standing challenge in cancer biology. Although GRNs comprise thousands of genes, normal and tumor tissues occupy distinct, low-dimensional attractors in expression space, raising the possibility that targeting a few key genes could induce widespread transcriptional changes. We build on two previously developed concepts: 1) N- and T-markers (genes with exclusive expression intervals in normal or tumor samples, respectively), and 2) Gene Deregulation Networks (GDNs) – directed acyclic graphs inferred from expression data, in which a link from C to E indicates that a deregulation at C increases the probability of a deregulation at E. A subset of N and T-genes, namely, NT-markers, appear in both networks and may act as bridges for phenotype reversal.

**Methods:**

Using TCGA bulk RNA-Seq data from five cancer types we identified N-, T- and NT-genes based on statistically significant exclusive expression intervals. Discretized expression states (N-active, T-active, inactive) are then associated with normal-exclusive, tumor-exclusive and non-exclusive expression intervals. GDNs were constructed with the CChains algorithm using the Loevinger coefficient, followed by Reichenbach (common cause) and Mokken (transitivity) pruning. We introduced a quantitative model to predict intervention outcomes in a tumor using gene activation frequencies and two topological metrics: the composed coverage and the fraction of the T-network unreached by the reverse deactivation cascade (U). We also use a Glauber-like dynamical model to simulate interventions.

**Results:**

We predict that pure T-gene interventions mainly alter the T-network, while pure N-gene interventions create a mixed normal-tumor state. In contrast, high-frequency NT-genes are predicted to simultaneously deactivate T-cascades and reactivate N-programs. The unreached fraction U varies across cancers: for perfect diagnostic panels, U ≈ 30% in PRAD but only ≈6% in LUAD. Escape probability also depends on tumor stage and on the spontaneous activation rate.

**Conclusion:**

NT-genes with high dual frequency are predicted optimal targets for partial phenotype reversal. The combination of composed coverage, unreached fraction and normal-state relevance provides a quantitative guide for designing multi-target therapies. The framework is general and yields testable predictions for intervention outcomes across cancer types.

## 1 Introduction

### 1.1 The long-standing goal of phenotype reversal

The idea of reverting a malignant cell to a normal phenotype has fascinated cancer researchers for decades (Telerman and Amson, 2009; Ingber, 2008; Huang and Kauffman, 2013). Early work identified molecular programs of tumor reversion, and systems biology approaches have recently given this concept a new momentum (Tripathi et al., 2021; Shin and Cho, 2023; Pensotti et al., 2024). Although gene regulatory networks involve thousands of interacting nodes (Kauffman, 1971; Davidson and Levine, 2005), the transcriptional states of normal and tumor tissues are surprisingly low-dimensional, typically requiring fewer than ten dimensions to capture most of the variance (Gonzalez et al., 2023). This low dimensionality, together with the observation that small panels of genes can perfectly discriminate between normal and tumor samples (Gil et al., 2025; Gil et al., 2026), suggests that manipulating a limited set of key regulators might be sufficient to induce widespread transcriptional changes, potentially pushing a tumor back towards a normal state.

### 1.2 N, T and NT markers: a systematic classification of cancer-associated genes

A critical step towards this goal has been the development of a systematic classification of genes based on their expression patterns across large cohorts of normal and tumor samples. The concepts of N-genes, T-genes and NT-genes were recently introduced in Ref (Gil et al., 2025) and further developed in the companion paper (Gil et al., 2026). N-genes are defined by the existence of a normal-exclusive expression interval that is significantly populated (at least 5% of normal samples using a Fisher test with p < 0.01). T-genes are defined by a tumor-exclusive expression interval with the same Fisher test (at least 10% of tumor samples). NT-genes satisfy both criteria. This classification goes beyond classical tumor suppressors and oncogenes (Dakal et al., 2024) because it captures not only mean shifts but also cases where a gene’s expression range in tumors lies completely outside the normal range (either above, below, or even both–what we called tumor-outside genes). Using this framework, we have shown that the number of active N-genes in normal samples decreases continuously as the tissue undergoes somatic evolution toward the tumor attractor, while the number of active T-genes increases continuously during tumor progression (Gil et al., 2026). This result is understood as a coarse-grained dynamics of cancer.

### 1.3 Gene Deregulation Networks (GDNs): a causal framework for intervention analysis

Building on this marker classification, a second companion paper (Castel et al., 2026) constructs Gene Deregulation Networks (GDNs) from the same TCGA data. GDNs are directed acyclic graphs in which a link from gene C to gene E indicates that a deregulation at C increases the probability of a deregulation at E, according to a probabilistic theory of causation (Suppes–Reichenbach). Separate networks are built for normal tissue (N-GDN) using inactive states as deregulation instances of N-genes, and for tumors (T-GDN) using active states as deregulation instances of T-genes. In prostate adenocarcinoma (PRAD), the N-GDN exhibits 492 representative nodes. In contrast, the T-GDN is much larger and sparser. Projecting individual tumor samples onto the T-GDN revealed that early tumors rely mostly on spontaneous activations (genes with no active parent), whereas advanced tumors show extensive cascade propagation (many genes activated through network edges). The equality between the Loevinger coefficients for direct and reverse processes suggested that forced deactivation of a T-gene propagates along reverse edges of the T-GDN, in the direction of lower frequencies, while forced activation of an N-gene propagates also along the reverse edges of N-GDN, but in the direction of increasing frequencies (decreasing deactivation frequencies). Importantly, we showed that an exhaustive diagnostic panel (the 8-gene T-panel with 100% sensitivity and 100% specificity in the training set in PRAD) is not necessarily an optimal therapeutic panel, because the reverse cascade from these genes may not reach the entire network, about 30% of the nodes of the T-GDN remain unreached, providing escape routes.

### 1.4 The gap: from markers and networks to target prediction for reversal

Despite these advances, neither of the preceding papers nor any paper in the literature, to the best of our knowledge, systematically address from a general perspective the central question of which genes are able to push a tumor back towards a mixed state with at least a partial restoration of normal-like programs.

In our framework, a tumor cannot have active N-markers. N-markers are characteristic of the normal state. In (Gil et al., 2026). we showed that even a single N-gene may sustain the normal-state condition. Then, if as a consequence of an intervention some N-genes are activated in a tumor, we define the result as a mixed normal-tumor state. The number of activated genes indicate the degree of restoration of normal programs. The frequencies of these genes are also important.

An intervention looking for tumor phenotype reversal should try to block the highest possible number of tumor programs and restore the highest possible number of normal programs. In other words: try to deactivate the highest possible number of active T-genes in the tumor and activate as much as possible N-genes. In the latter case, it is not completely clear for us whether the number of N-genes or their composed coverage is the key factor in restoring N-programs.

### 1.5 Novel contributions of the present work

The present work fills this gap by combining the N/T/NT marker framework with GDN topology to develop a predictive, clinically relevant synthesis.

Our novel contributions are threefold. First, we introduce static topological metrics–namely, the composed coverage (F_T_) and the unreached fraction (U) – that allow a rapid, simulation-free assessment of the intervention potential. Second, we demonstrate theoretically that high-frequency NT-genes are uniquely positioned to induce partial phenotype reversal, because they simultaneously induce T-gene deactivation cascades and reactivate N-programs. Third, we show that escape probability depends not only on U but also on tumor stage and the spontaneous activation rate, providing a more complete picture of therapeutic resistance.

Our framework thus provides a practical, quantitative guide for prioritizing gene therapy targets and designing combination strategies, with clear testable predictions for future experiments.

### 1.6 Summary of existing computational methods for predicting effects of genetic perturbations

Predicting the transcriptional consequences of genetic perturbations—such as gene silencing or overexpression—is a well-established challenge in bioinformatics. However, unlike the majority of contemporary tools that are exclusively designed for single-cell RNA-seq data, we shall focus on methods that are either built for, or readily applicable to, bulk RNA-seq data. Among them, we may distinguish deep learning models, causal inference frameworks, and, notably, simple baseline estimates that often prove surprisingly competitive.

The Large Perturbation Model (LPM) is a deep learning framework which integrates heterogeneous perturbation experiments—including both single-cell and bulk data—by representing the perturbation, the readout type, and the biological context as separate dimensions, enabling the prediction of transcriptomes for unseen experimental conditions (Miladinovic et al., 2025).

BulkFormer, positioned as a large-scale foundational model, comprises 150 million parameters and is pre-trained on over 500,000 human bulk transcriptome profiles, making it a powerful resource for predicting cellular states from bulk measurements (Kang et al., 2025).

The SIDISH framework employs a neural network architecture that integrates the granularity of single-cell data with the scalability of bulk RNA-seq using a variational autoencoder, effectively bridging the resolution gap between the two technologies (Jolasun et al., 2025).

TxPert is a deep learning method based on latent transfer learning, which leverages multiple biological knowledge graphs to predict transcriptomic perturbation outcomes (Wenkel et al., 2026). Its flexible architecture allows for application to bulk-level predictions.

Diverging from standard neural networks, CIPHER applies the linear response theory from statistical physics to predict whole-transcriptome perturbation outcomes by leveraging gene co-fluctuation patterns observed in unperturbed cells (Kuznets-Speck et al., 2025).

The Root Causal Strength using Perturbations (RCSP) method estimates the root causal strength (RCS) of genes directly from bulk RNA-seq data. It learns the causal ordering of genes using Perturb-seq data and transfers this order to bulk samples to identify patient-specific causal driver genes (Strobl and Gamazon, 2025).

On the other hand, a critical insight emerging from recent large-scale evaluations is the remarkable efficacy of simple approaches. A pivotal 2025 study published in Nature Methods benchmarked five foundational models and two additional deep learning architectures against simple linear baselines for predicting transcriptomic changes (Ahlmann-Elze et al., 2025). The key finding was that none of the complex models outperformed these elementary baselines. Additive models (which predict the effect of a double perturbation as the sum of individual effects) and simple models based solely on gene expression (such as Pearson correlation or GRNBoost2) have consistently been shown to match or exceed the performance of more intricate inference networks in benchmarking studies.

We stress that while most existing methods operate as black boxes or output continuous expression scores, our framework introduces biologically interpretable, quantifiable metrics specifically designed for combination therapy optimization. These metrics provide actionable guidance for multi-target selection via Pareto optimization.

On the other hand, our reliance on simple statistical measures—activation frequencies and network propagation rules—is aligned with the empirical findings of the Nature Methods (2025) study (Ahlmann-Elze et al., 2025). While deep learning models demand extensive computational resources, large-scale training data, and complex hyperparameter tuning, our framework is computationally lightweight, easy to implement, and highly interpretable. It does not require large-scale perturbation training sets, making it accessible to a broad research community without sacrificing predictive relevance.

## 2 Methods

### 2.1 Data acquisition and definition of N-, T- and NT-genes

We used TCGA level 3 RNA-Seq expression data (FPKM units) for five cancer localizations: prostate adenocarcinoma (PRAD (Cancer Genome Atlas Re search Network, 2015)), head and neck squamous carcinoma (HNSC (Cancer Genome Atlas Network, 2015)), lung adenocarcinoma (LUAD (Cancer Genome Atlas Research Network, 2014)), uterine corpus endometrial carcinoma (UCEC (Cancer Genome Atlas Research Network et al., 2013)), and lung squamous cell carcinoma (LUSC (Cancer Genome Atlas Research Network, 2012)). Sample counts were as follows: PRAD (52 normal, 499 tumor), HNSC (44 normal, 502 tumor), LUAD (59 normal, 535 tumor), UCEC (23 normal, 552 tumor), LUSC (49 normal, 502 tumor).

Following the procedure established in (Gil et al., 2026), a gene was classified as an N-gene if there existed a normal-exclusive expression interval containing at least 5% of normal samples (one-sided Fisher test, p < 0.01). A T-gene, with the same test, required a tumor-exclusive interval containing at least 10% of tumor samples. NT-genes satisfied both criteria. The remaining genes (neither normal-exclusive nor tumor-exclusive intervals) were termed O-genes and were not further considered in the dynamics. In PRAD, this yielded 1097 N-genes, 6138 T-genes, and 316 NT-genes.

Expression was discretized into three states: e = −1 (N-active) if the expression fell within a normal-exclusive interval; e = +1 (T-active) if it fell within a tumor-exclusive interval; and e = 0 (inactive) otherwise. Pure N-genes can only take values −1 or 0; pure T-genes only +1 or 0; NT-genes can take any of the three states. Activation frequencies f_N_ and f_T_ were defined as the proportions of normal or tumor samples, respectively, in which the gene was N-active or T-active.

### 2.2 Gene Deregulation Networks (GDNs)

GDNs were constructed as described in Ref (Castel et al., 2026). using the CChains algorithm (https://github.com/gabriel-gil/CChains). For each tissue type, we built two binary matrices. For the N-GDN, rows corresponded to N-genes, columns to normal samples, and an entry was 1 if the N-gene was deregulated (i.e., e = 0, inactive, meaning that the gene had lost its N-active state). For the T-GDN, rows corresponded to T-genes, columns to tumor samples, and an entry was 1 if the T-gene was deregulated (i.e., e = +1, active).

The algorithm then proceeded through four stages: (i) Loevinger coefficient with threshold H_0_ = 0.5 to declare *prima facie* causal relations; (ii) Reichenbach pruning to remove common-cause artifacts; (iii) Mokken pruning to remove transitive edges; and (iv) orientation of remaining undirected edges using a Mokken-based heuristic. The resulting networks were directed acyclic graphs (DAGs). For the N-GDN, genes with identical deregulation profiles across all samples were compressed into blocks, each block represented by a single node. In PRAD, the largest block contained 216 genes, reducing the N-GDN from 1097 to 492 representative nodes.

### 2.3 Dynamical rules for interventions

The dynamics for interventions were taken directly from (Castel et al., 2026). Let us stress that they are not empirically established rules, but rules following from our theoretical scheme that lead to testable predictions.

In a tumor, spontaneous evolution involves the progressive activation of T-genes, propagating in the direction of the T-GDN edges. Forced deactivation of a T-gene propagates against the direction of the edges (reverse cascade). For a normal tissue, spontaneous evolution involves the progressive deactivation of N-genes; forced activation of an N-gene propagates along the reverse N-GDN edges (i.e., towards higher frequencies). For NT-genes, forcing the N-active state in a tumor triggers both a deactivation cascade in the T-GDN (reverse edges) and an activation cascade in the N-GDN (reverse edges).

These rules were implemented in a Glauber-like discrete-time Monte Carlo simulation with spontaneous activation probability p_spont_ (typically 0.1) and forced deactivation probability p_forced_ (typically 0.5). Once a gene was deactivated, it could not be reactivated.

### 2.4 Metrics for predicting intervention outcome

We introduced two static topological metrics.

The first is the composed coverage: for a set of target genes {g_1_, g_2_, … }, F_T_ = f_T_(g_1_ ∨ g_2_ ∨ …), i.e., the fraction of tumor samples in which at least one target is T-active. The symbol∨is the logical OR.

The second is the unreached fraction U of the T-network: starting from the target gene(s), we compute the set of T-genes reachable by following reverse edges (parents, then parents of parents, etc.) in the T-GDN. The fraction of all T-nodes not reachable by this reverse cascade is U. This provides a structural lower bound for potential escape routes. Simulations also incorporated tumor stage (initial number of active T-genes) and the spontaneous activation rate p_spont_.

### 2.5 The need of experimental validation

Our framework, based on N- and T-genes and deregulation networks, is completely novel. A systematic validation, although necessary, is beyond the scope of the present work. In Ref. (Castel et al., 2026), we preliminarily analyzed an experiment on the knockdown of the POM121 gene in two prostate cancer cell lines (Kirthika et al., 2025). The sample sizes in this study are relatively small and insufficient to define N- and T-genes from scratch. Therefore, we rely on TCGA data to obtain the gene lists and compare with the TCGA mean log-fold value in order to decide whether a gene is activated or deactivated after intervention.

In Supplementary Section S1 of the Supplementary Material we refine the algorithm to decide whether the experimental log-fold reported for a given gene correspond to an activation or deactivation event. Admittedly, this procedure introduces batch effects (experimental data versus TCGA). We assume that log-fold variations are less sensitive to batch effects.

In Supplementary Section S1 we reanalyze the example treated in (Castel et al., 2026), and add two new examples involving NT genes: knockdown of the FGFRL1 gene in the PC3M cell line (Yu et al., 2022), and knockdown/overexpression of the YAP1 gene in three prostatic cancer cell lines with the purpose of restoring/reversing the neuroendocrine phenotype (Asrani et al., 2021).

In each of the studied examples we shall report.

1. Probability of activation/deactivation of the target gene. This is an important quantity because sometimes it is not experimentally achieved. In case of deactivation of an NT-gene in a tumor, we evaluate also its possible N-activation, that is whether an N-cascade is induced.
2. The induced activation/deactivation cascade. That is: number of deactivated/activated genes and total number of genes in the cascade depth.
3. The number of non-cascade activated/deactivated genes and the total number of measured genes.

The examples show that the results depend on the degree of activation/deactivation of the target gene, on the frequency of the target gene, and on the time where measurements were performed. It seems that experiments are most probably reporting transient results. A high number of observed spontaneous activation/deactivation events is probably elicited by the intervention.

The new examples are sourced from the PerturbAtlas portal (https://perturbatlas.kratoss.site (Zhang et al., 2025)), which hosts approximately 1,290 cancer-related perturbation studies with bulk RNAseq measurements. A systematic analysis of all cancer data from the platform is in course and will be reported elsewhere.

## 3 Results

### 3.1 Qualitative prediction of single-gene interventions

**FIGURE 1:**
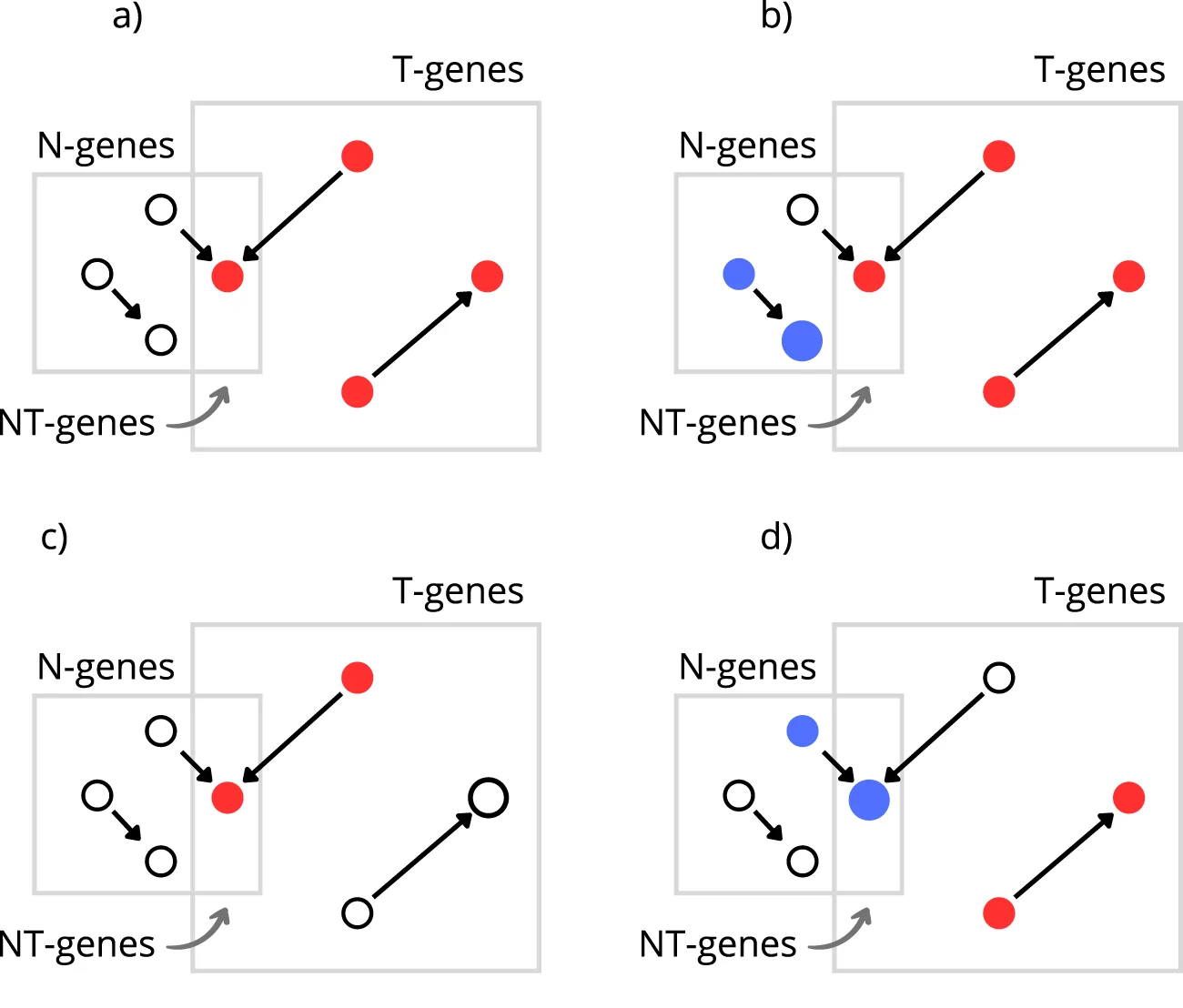
Schematic representation of single-gene intervention outcomes. Colors define the activation state: red–T-active, blue–N-active, white–Deactivated. Arrows indicate causal links in the corresponding GDN. The perturbed gene is marked with a bigger symbol. **(a)** Initial tumor state. **(b)** Intervention on pure N-gene: mixed state (partial reversal). **(c)** Intervention on pure T-gene: modified tumor state. **(d)** Intervention on NT-gene: mixed state with dual cascade.

Figure 1 (see caption) provides a schematic representation of the possible outcomes of a single-gene intervention in a tumor, based on the network architecture and the dynamical rules described above. Three distinct scenarios are predicted.

Case (b): Forcing the activation of a pure N-gene in a tumor creates a mixed state in which some N-markers become active while pre-existing T-markers remain active. This represents a partial phenotype reversal–the cell population exhibits features of both normal and tumor states. The intervention goes against the spontaneous evolution of the N-network, and forced activation propagates backward along the N-GDN edges, activating other N-genes with higher f_N_. However, no direct deactivation of T-genes occurs unless NT-genes mediate. Therefore, the mixed state is achieved by adding normal features without removing tumor features.

Case (c): Forcing the deactivation of a pure T-gene propagates along reverse edges of the T-GDN, deactivating upstream (lower-frequency) T-genes. This modifies the tumor state, potentially reducing its malignancy, but it does not, by itself, reactivate normal programs. A mixed state may occur only if the targeted T-gene (or genes in its reverse cascade) is connected to the N-network through NT-genes. In the absence of such connections, the outcome is a modified tumor state, not a reversal. This case likely corresponds to the typical outcome of many targeted therapies.

Case (d): Forcing an NT-gene into its N-active state (e = −1) triggers two simultaneous cascades. On the T-GDN side, because the NT-gene is also a T-gene, forcing it to e = −1 means it is no longer T-active; this deactivation propagates along reverse edges, deactivating upstream T-genes (those with lower f_T_). On the N-GDN side, the forced N-activation propagates forward along N-edges, activating upstream N-genes (those with higher f_N_). The result is a mixed state that actively counteracts both the spontaneous tumor progression (by deactivating T-cascades) and the loss of normal identity (by reactivating N-cascades). This dual effect makes NT-genes uniquely positioned to induce true phenotype reversal.

### 3.2 *AGER* and other examples of NT-genes in different tumor localizations

Lung adenocarcinoma provides a preliminary experimental validation of the result of intervening an NT-gene (case d). The gene *AGER* (advanced glycosylation end-product specific receptor) is an NT-gene with f_T_ = 0.98 (active in 98% of LUAD tumors) and f_N_ = 0.75 (active in 75% of normal lung samples). *AGER* is a normal-above/tumor-below NT-gene: in normal samples, high expression is N-active; in tumors, low expression is T-active.

**FIGURE 2:**
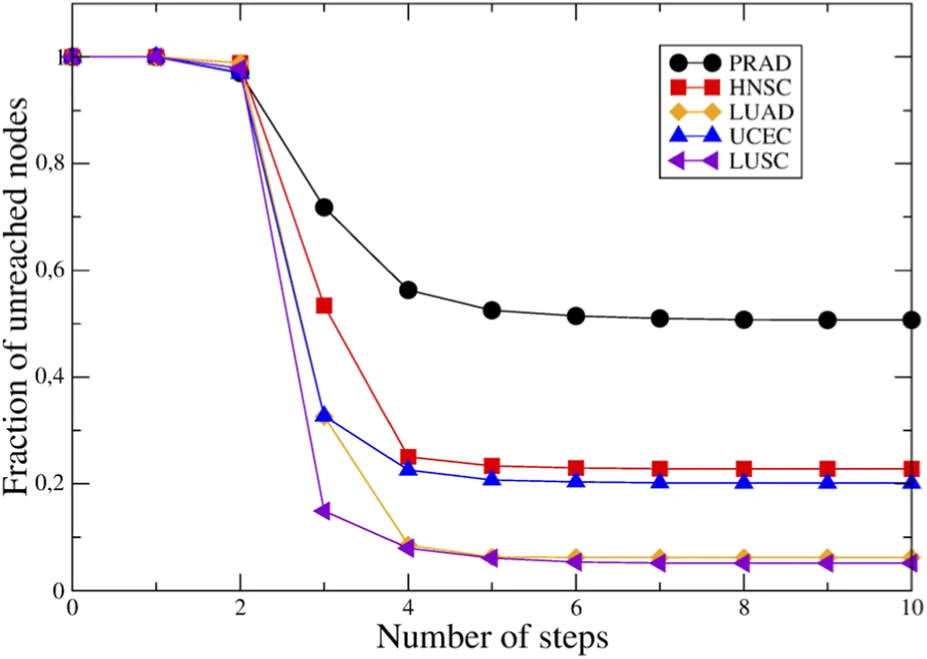
Fraction of T-network unreached by the reverse cascade from the highest-frequency T-gene in five cancer types (PRAD, HNSC, LUAD, UCEC, LUSC) as a function of cascade depth.

In the T-GDN of LUAD, *AGER* is the highest-frequency node. Forcing it into its N-active state triggers a reverse deactivation cascade that reaches more than 90% of the T-network, leaving U ≈ 10% (as shown in Figure 2). On the N-GDN side, forcing *AGER* into N-active state triggers a forward activation cascade, but only genes with higher f_N_ than *AGER* are reachable–in LUAD, only *STX11* (f_N_ = 0.81) satisfies this condition. However, *AGER* and *STX11* are the highest frequency N-genes and are thus strongly related to the normal phenotype. On the other hand, 387 of the 492 N-genes in LUAD are actually NT-genes. A large fraction of the N-network, in principle, could be indirectly affected through the T-network deactivation cascade.

Experimental work by Wang et al. (Wang et al., 2020) provides preliminary support. They overexpressed *AGER* in H1299 non-small cell lung cancer cells. Overexpression significantly inhibited cell proliferation, migration, and invasion, induced apoptosis, and partially restored a normal-like epithelial phenotype. Conversely, knocking down *AGER* induced tumor-associated changes. This bidirectional control–shifting cells between normal and tumor-associated states by manipulating a single NT-gene–is exactly what our framework predicts. Thus, *AGER* represents a prime candidate for therapeutic intervention aimed at partial phenotype reversal in LUAD. Let us stress that the required degree of overexpression is determined experimentally by comparing with a normal tissue cell line.

The *AGER* gene is ranked second among NT-targets in lung adenocarcinoma (Gonzalez et al., 2025). Numerous experiments support the relevance of high-frequency NT-genes in other tissues, and we briefly examine some examples.

The effects of modulating the scavenger receptor class A member 5 gene (*SCARA5*) in four colorectal cancer cell lines were studied in (Li et al., 2021). *SCARA5* is a perfect NT-gene in our scheme (f_T_ = f_N_ = 1) and is ranked first among single targets in this tissue (Gonzalez et al., 2025).

The *VSTM2A* gene (V-set and transmembrane domain containing 2A) is also among the top-ranked genes in colon (f_T_ = 0.96, f_N_ = 0.85). Its relevance, related to its role in immune suppression, was investigated in (Dong et al., 2024) using a mouse model. This example stresses the ability of our scheme to identify targets playing a role in the tumor microenvironment.

The vascular endothelial growth factor D gene (*VEGFD*) is ranked first among NT-genes in breast carcinoma (f_T_ = 0.93, f_N_ = 0.45) (Gonzalez et al., 2025). Its relevance was investigated in breast cancer cell lines and a mouse model (Akahane et al., 2005).

The inter-α-trypsin inhibitor heavy chain 5 gene (*ITIH5*) is ranked first for bladder cancer (f_T_ = 0.87, f_N_ = 0.47) (Gonzalez et al., 2025). In Ref. (Rose et al., 2014), forced expression of *ITIH5* in RT112 bladder cancer cells was shown to lead to suppression of cell migration and inhibition of colony spreading, thereby providing evidence that downregulation of *ITIH5* by aberrant DNA hypermethylation may provoke invasive phenotypes in bladder cancer.

The *TOMM40* gene, encoding an outer membrane channel protein essential for maintaining mitochondrial function, is ranked first for cervical squamous carcinoma (f_T_ = f_N_ = 1) (Gonzalez et al., 2025). Modulation of this gene was investigated in Ref (Yang et al., 2020). using an epithelial ovarian cancer cell line and a xenograft mouse model, and the results confirm its relevance.

The vacuolar protein sorting 45 (*VPS45*) gene is ranked second for hepatocellular carcinoma (f_T_ = 0.89, f_N_ = 0.74) (Gonzalez et al., 2025). Its relevance was investigated recently in (Zhi et al., 2025). The authors verified the bidirectionality of changes in proliferation, invasion, and migration by manipulating gene expression levels in two cancer cell lines and normal hepatocytes.

The myeloid-specific hematopoietic cell kinase gene (*HCK*) is ranked second in pancreatic adenocarcinoma (f_T_ = 0.97, f_N_ = 0.50) (Gonzalez et al., 2025). In Ref. (Poh et al., 2022), the authors used a xenograft model to confirm HCK as a critical promoter of myeloid-mediated immune suppression, metastasis, and desmoplasia in pancreatic cancer.

The *SH3GLB1* gene, encoding Endophilin B1, is ranked first in prostate cancer (f_T_ = 0.58, f_N_ = 0.25) (Gonzalez et al., 2025). In Ref. (Zhu et al., 2016), studies in 2 cell lines demonstrate that Endophilin B1 controls proliferation through its action on EGFR.

These examples provide evidence for the relevance of NT-genes in cancer. In some cases, there is even demonstration of bidirectional phenotype control. However, direct evidence of the reverse cascades in the T- and N-GDNs is still lacking and requires further validation.

### 3.3 Escape under single-gene interventions in tumors with different levels of transcriptional re-programming

Even after a successful intervention, residual tumor cells may harbor pathways that enable escape and disease progression. To understand this process, we examined the structural features of the T-GDN that foster escape. In particular, we evaluated the fraction of the T-network that remains unreached (U) by a deactivation cascade starting at the highest-frequency T-gene for each of the five cancer types (PRAD, HNSC, LUAD, UCEC, LUSC). Figure 2 shows the fraction of unreached nodes as a function of the depth of the reverse cascade (number of steps). At the first step, we reach only the parents of the intervened gene; at the second step, the parents of those parents, and so on.

For prostate adenocarcinoma (the most heterogeneous cancer in our set), the cascade reaches almost 50% of the network, leaving about 50% unreached even after many steps. For the two lung cancer types (LUAD and LUSC), the cascade reaches about 90% of the network, leaving only about 10% unreached. The other cancer types (HNSC, UCEC) show intermediate behaviour. These differences reflect the underlying network topology and the hierarchical position of the highest-frequency gene in each cancer. Importantly, the unreached fraction sets a structural lower bound for escape: even under ideal conditions, genes in the unreached region cannot be directly deactivated by the intervention and may eventually drive relapse. This analysis demonstrates that single-gene interventions, even against the most frequent T-gene, leave a substantial escape reservoir in heterogeneous tumors like PRAD, whereas in less heterogeneous tumors like LUAD the reservoir is much smaller.

As an example, we may evaluate the coverage and unreached fraction of the *EPHA2* gene across the five tumors under study. A vaccine for solid tumors based on a toxin targeting this gene has been tested recently (Bashir et al., 2024). The cohort includes five tumor localizations, in particular a small number of lung cancer patients.

The results of our evaluation are shown in Table 1. EPHA2 is not identified as a T-gene in three tumors, indicating the need for tumor-oriented, not universal, targets. In addition, in PRAD and UCEC it is a low-frequency high-U target. We predict a low-efficiency vaccine in these tumors.

**TABLE 1:** Coverage and unreached fraction for single targets and combinations across the five tumors under study. Not reported values indicate that the target was not a T-gene in that tumor. Values are rounded to three decimal places.

Target gene	PRAD	HNSC	LUAD	UCEC	LUSC
EPHA2					
Coverage	0.250	---	---	0.107	---
Unreached fraction	0.945	---	---	1.000	---
TGFB1					
Coverage	---	0.627	0.222	0.120	0.349
Unreached fraction	---	0.969	0.998	1.000	1.000
PTGS2					
Coverage	0.224	0.195	0.237	0.302	0.335
Unreached fraction	0.995	1.000	1.000	1.000	1.000
TGFB1 & PTGS2					
Coverage	0.224	0.705	0.402	0.377	0.558
Unreached fraction	0.995	0.969	0.998	1.000	1.000

### 3.4 Multi-target interventions: balancing coverage and escape

When combining multiple T-gene targets, two objectives must be balanced. The first is coverage: the fraction of tumor samples in which at least one target is T-active, denoted F_T_ = f_T_(g_1_ ∨ g_2_ ∨ …). This is exactly the sensitivity of a diagnostic panel based on those genes.

The second is minimizing escape: reducing the fraction of the T-network that remains unreached (U) by the reverse deactivation cascade starting from the targets. Unreached genes cannot be directly deactivated and may serve as seeds for new activation cascades.

**FIGURE 3:**
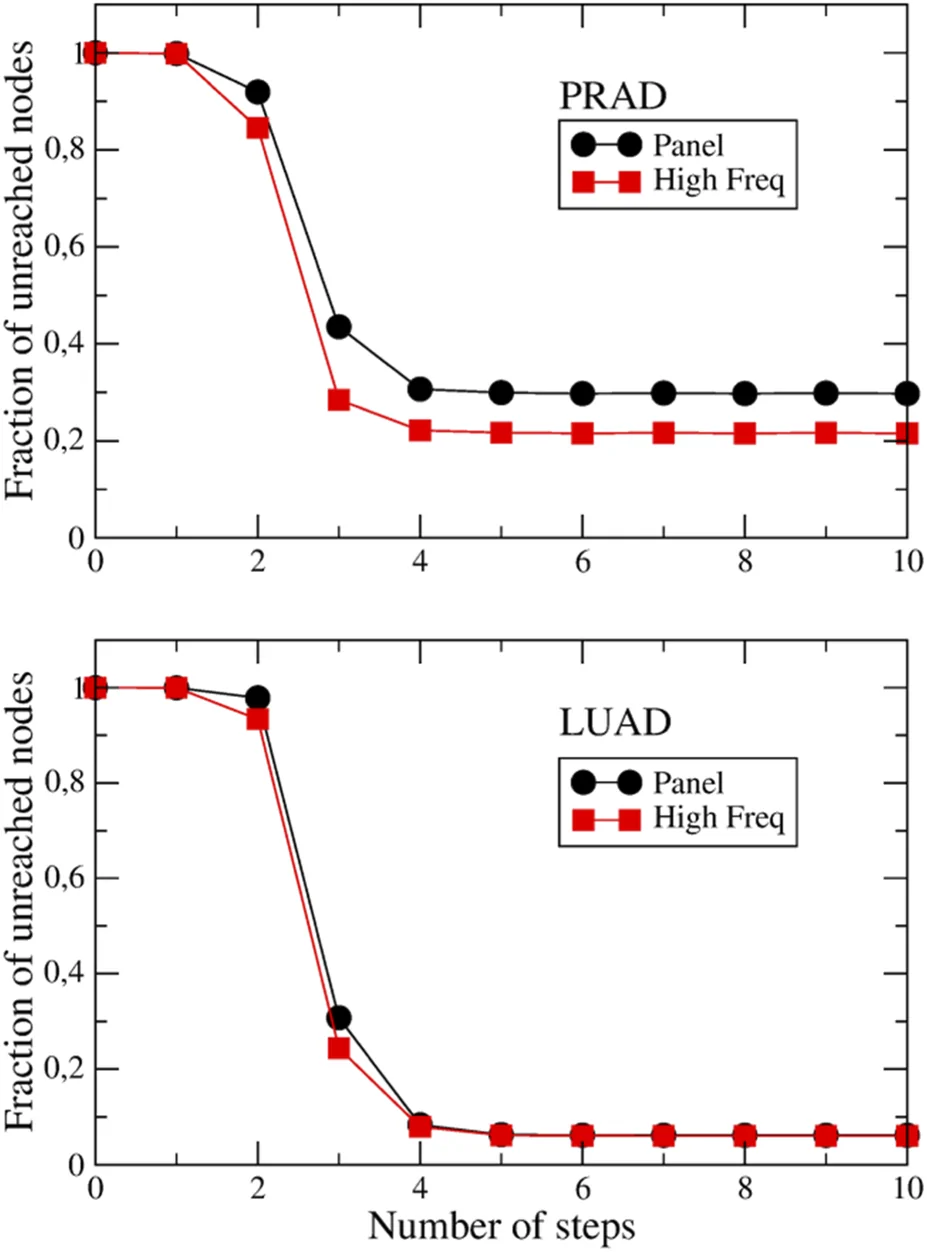
Unreached fraction U for different target sets. Top: PRAD–perfect 8-gene panel (black circles) vs. Eight highest-frequency T-genes (red squares). Bottom: LUAD–perfect 2-gene panel (black circles) vs. Two highest-frequency T-genes (red squares).

To illustrate this trade-off, we considered prostate adenocarcinoma (PRAD) and lung adenocarcinoma (LUAD). In PRAD, the perfect diagnostic panel of eight genes (EPHA10, UCN, TMEM86A, RPL26P23, RBM15B, NPY4R2, TEKT4, ENSG00000271895) achieves F_T_ = 1.00, meaning that at least one gene in this panel is active in every tumor sample. However, the reverse cascade starting from these eight genes leaves U ≈ 30% of T-nodes unreached (Figure 3, top panel). In contrast, a set of the eight highest-frequency T-genes (ranked by f_T_, regardless of whether they form a perfect panel) gives F_T_ = 0.69 (i.e., 31% of tumors have none of these genes active) but reduces U to ≈20% (Figure 3, top panel). Thus, sacrificing full coverage reduces the escape reservoir. This shows that a perfect diagnostic panel is not necessarily a perfect therapeutic panel.

In LUAD, the distance between the normal and tumor attractors is larger, and the tumor is less heterogeneous. The perfect diagnostic panel contains only two genes: *AGER* and *SLC37A4*, with F_T_ = 1.00. For these two genes, U ≈ 6% (Figure 3, bottom panel). The set of the two highest-frequency T-genes (which includes *AGER* and another high-frequency gene) gives similar coverage (>98%) and similar U (≈6%). Therefore, in LUAD there is almost no trade-off: high coverage and low escape can be achieved simultaneously. Cancers with more distant attractors (higher transcriptional reprogramming) may be more amenable to targeted reversal.

Also as an example, we examine the combination of two targets used in the clinical trial NCT04669808 (Clinicaltrials, 2025). The targeted genes are TGFB1 and PTGS2 (COX-2) and the tumor under study is basal cell carcinoma. Unfortunately, there are not data for basal cell carcinoma in the TCGA portal. Thus, we evaluate coverage and the unreached fraction for each gene and their combination in the five tumors we study. Results are reported in Table 1 also.

Except for the *TGFB1* gene in PRAD, all genes are identified as T-genes across the five tumors. The combination coverage ranges from 0.22 in PRAD to 0.77 in HNSC. However, the U-fraction remains very high. Our prediction is that the vaccine would be largely ineffective in these tissues.

The examples presented in Sections 3.3 and 3.4 can be regarded as *in silico* analyses evaluating the possibility of repurposing existing vaccines. They illustrate the ability of our metrics to evaluate the effects of intervening single targets and combinations across different tissues.

### 3.5 Escape probability depends on tumor stage and activation rate

The fraction of the T-network that remains unreached (U) sets a lower bound on the set of genes that the tumor may use as escape routes. However, two additional factors determine escape after intervention: the initial tumor stage (number of active T-genes) and the spontaneous activation rate of new T-genes.

**FIGURE 4:**
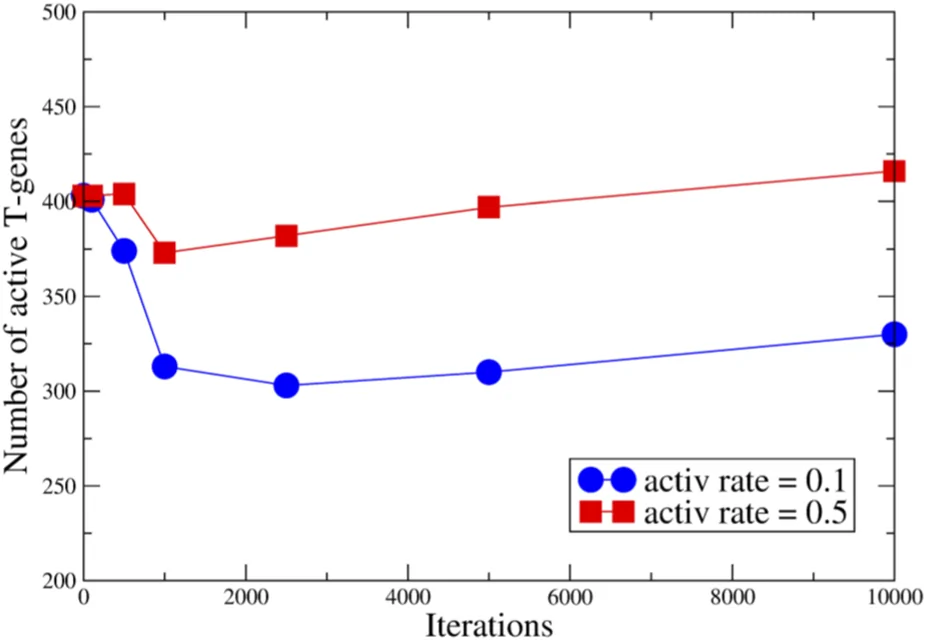
Pseudotime evolution of active T-genes after deactivating the perfect panel in a young PRAD tumor (403 active genes). p_forced_ = 0.5; p_spont_ = 0.1 (slow recovery) vs. 0.5 (fast recovery, escape after ∼8000 steps).

We performed simulations following the procedure described in Methods. The deactivation rate (p_forced_) was set to 0.5. First, we considered a relatively young PRAD tumor sample with 403 active T-genes (this sample lies near the diagonal in the spontaneous vs. cascade activation plot, indicating that most activations are spontaneous). The whole perfect panel of eight genes was forcibly deactivated. The unreached fraction U for this panel is about 30%. We then ran simulations for two values of the spontaneous activation rate p_spont_: 0.1 (slow activation) and 0.5 (fast activation, equal to the deactivation rate). The results are shown in Figure 4.

When p_spont_ = 0.1, the number of active T-genes dropped from 403 to about 200 and remained low for thousands of iterations, indicating a durable response. When p_spont_ = 0.5, the tumor recovered: after about 8,000 iterations, the number of active T-genes returned to the initial level (403). Recovery occurred because the unreached nodes (30% of the network) could be spontaneously activated at the same rate as they were being deactivated, and cascades from these reactivated nodes eventually repopulated the network.

In contrast, for a very advanced tumor (e.g., a sample with 2,640 active T-genes, lying far above the diagonal, indicating extensive cascade propagation), the same intervention led to a monotonic decrease in active T-genes that never recovered, regardless of p_spont_. This is because most of the active genes in an advanced tumor are already part of long cascades and are downstream of many parents; deactivating the perfect panel and its reverse cascade removes a large fraction of the network, and the unreached reservoir is not sufficient to re-establish the original high activity level.

We therefore conclude that three factors jointly determine escape after intervention: the structural unreached fraction U (determined by the target set and network topology), the initial tumor stage (number of active T-genes), and the spontaneous activation rate p_spont_. Controlling the activation rate–for example, by combining gene therapy with conventional cytotoxic agents that reduce cell division–should be a central goal of combination therapy.

### 3.6 Multi-objective optimization for NT-target combinations

For interventions targeting NT-genes, an additional criterion must be considered: the frequency in normal samples, f_N_. Because NT-genes are active in normal tissues, those with high f_N_ are most relevant for normal tissue identity. When forcing an NT-gene into its N-active state, a higher f_N_ likely means a stronger restoration of normal programs. Therefore, for NT targets, we should maximize F_T_ (coverage), minimize U (escape), and maximize f_N_ (normal relevance). This three-dimensional optimization can be addressed with Pareto-based methods (Paquete, 2006). A method for selecting such optimal targets has been described in a pending patent application (Gonzalez et al., 2025) and will be the subject of future work.

## 4 Discussion

**FIGURE 5:**
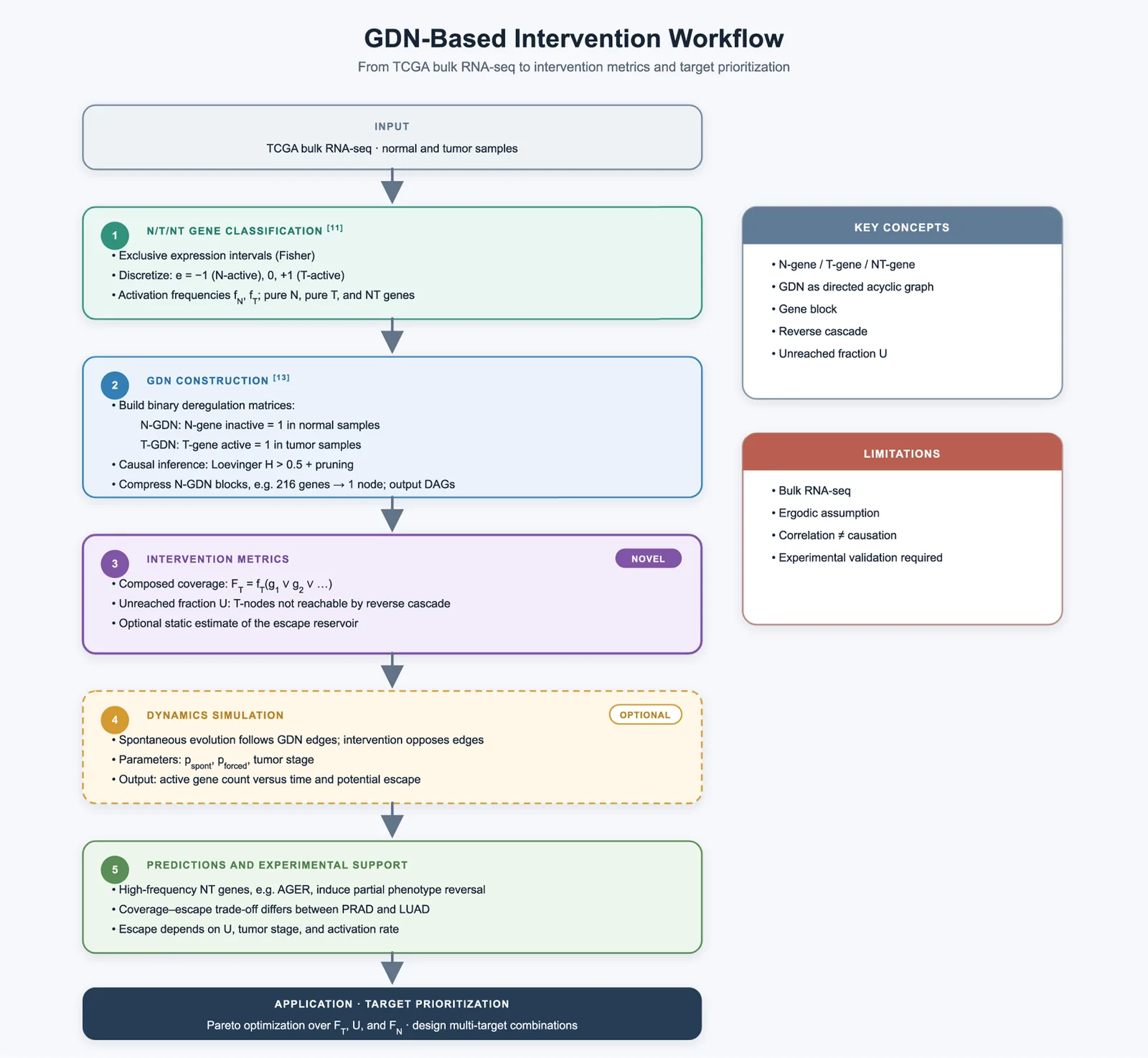
Schematic workflow of the proposed framework for predicting intervention outcomes and tumor escape. The diagram shows the sequential steps from TCGA data input (top) to final target prioritization (bottom). Key stages include: N/T/NT gene classification using exclusive expression intervals and frequency calculation (Gil et al., 2026); construction of Gene Deregulation Networks (GDNs) as directed acyclic graphs via causal inference (Castel et al., 2026); introduction of novel static metrics (composed coverage F_T_ and unreached fraction U); optional dynamics simulation; experimental validations; and final multi-objective optimization for target selection. Side boxes highlight key concepts and limitations.

Figure 5 provides a schematic workflow summarizing the entire framework, from data input to the final predictions and limitations.

### 4.1 Summary of contributions and novelty

In this work we have developed a quantitative, network-based framework to predict the outcomes of gene interventions aimed at reversing the tumor phenotype. The framework builds directly on the N/T/NT marker classification and the GDNs from our two precedent papers (Gil et al., 2026; Castel et al., 2026), but it goes beyond them by providing a predictive synthesis focused on phenotype reversal–a question that was not systematically addressed before. Our novel contributions include the introduction of static metrics (F_T_ and U), the demonstration that high-frequency NT-genes are optimal for partial reversal and the identification of three factors governing escape (U, tumor stage, activation rate).

### 4.2 Why NT-genes are optimal for reversal

The central insight is that NT-genes occupy a privileged position at the interface of normal and tumor networks. Their high frequencies in both states (e.g., AGER: f_T_ = 0.98, f_N_ = 0.75) indicate that they are central to tissue identity in both contexts. Interventions on NT-genes simultaneously deactivate T-cascades and reactivate N-programs (via reverse edges) – the dual requirement for true phenotype reversal. Experiments involving AGER and other NT-genes in different tissues provide preliminary experimental support. To our knowledge, this is the first time the NT-gene concept has been explicitly linked to phenotype reversal in a predictive, experimentally testable manner.

### 4.3 The coverage-escape trade-off and its clinical implications

Our analysis reveals that a perfect diagnostic panel is not necessarily a perfect therapeutic panel. In PRAD, full coverage leaves 30% of the T-network unreachable, providing escape routes. In LUAD, the same trade-off is negligible. This suggests that cancers with more extensive transcriptional reprogramming may be more amenable to targeted reversal–a hypothesis that can be tested across other cancer types. Clinically, our framework provides a rationale for combining gene therapy with agents that reduce the spontaneous activation rate (e.g., proliferation inhibitors) to minimize escape.

### 4.4 Escape as a multi-factor process

Simulations show that escape is not determined solely by U. Young tumors are more plastic and can recover if the activation rate is high; advanced tumors are less plastic and can only become less malignant. This implies that the timing of intervention matters: early intervention may be more effective if the activation rate can be controlled, but late intervention may still reduce malignancy even if escape occurs. These insights go beyond static network analyses and provide a dynamical perspective on therapeutic resistance.

### 4.5 Missing experimental tests and future directions

The analysis of a few perturbation experiments measuring bulk RNAseq data does not validate nor disprove the T-GDN predictions, as shown in Supplementary Section S1 of Supplementary Material. The N-GDN predictions remain untested also. Examples targeting *AGER* or other NT-genes in different tumors show preliminary support, although a more systematic effort is needed.

Let us stress that some of our predictions could be straightforwardly tested. For example, targeting *AGER* in LUAD shall induce the N-activation of *STX11*. The combination *AGER* + *SLC37A4* is almost perfect in LUAD, leaving only a 6% probability of escape. Targeting high-frequency genes and their combinations in all tumors shall lead to much better results than targeting mutational drivers and other low-frequency genes.

Future work will also extend the framework to single-cell data, integrate perturbation screens to refine causal edges, and apply Pareto optimization to design optimal NT-target combinations.

## Data Availability

All data used in this study are publicly available through the TCGA Research Network (https://www.cancer.gov/tcga). The complete source code of CChains is available at https://github.com/gabriel-gil/CChains. A companion repository for the GDN framework is available at https://github.com/fcastelm/GDN. The binary deregulation matrix used as input by CChains is generated using the GenePan code at https://github.com/gabriel-gil/GenePan. The GeneGlauber code, to perform the Glauber dynamics, is available at https://github.com/gabriel-gil/GeneGlauber.
