# Supplementary material for: Bidirectional network hubs: NT-genes as candidate targets for partial cancer reversal
Source: Front Syst Biol. 2026 Sep 8;6:1868020. doi: 10.3389/fsysb.2026.1868020 (PMC13593772; doi:10.3389/fsysb.2026.1868020)
Supplement: Supplementary file 1 [file DataSheet1.pdf]

Bidirectional network hubs: NT-genes as optimal targets for partial cancer reversal

Gabriel Gil[1]*, Rolando Perez[2], Augusto Gonzalez[1]*

Supplementary Material

S1. Perturbation-response analysis in PRAD cell-line experiments

Analysis overview

We analyzed differential expression profiles from PRAD cell-line perturbation experiments by mapping measured genes to the PRAD TCGA expression dataset. N-, T-, and NT-gene discovery, family classification, and the construction of N- and T-GDNs were based exclusively on TCGA data. Only above and below N- and T-gene families were examined in this analysis. Each gene was assigned its corresponding TCGA-derived exclusive expression interval.

For each mapped gene measured in the experiment, TCGA samples were used to construct empirical distributions of log2 expression transitions from initial states compatible with the control phenotype. Because the control phenotype in these experiments is tumorous, the initial states can correspond to either a T-active or an inactive state, depending on the modification category being tested. For T-genes, T-activation was tested using transitions from inactive to T-active states, whereas T-deactivation was tested using transitions from T-active to non-T-active states. For N-genes, N-activation was tested using transitions from inactive or T-active states toward N-active states.

For each tested modification category (T-activation, T-deactivation, or N-activation), we first selected all transitions between TCGA sample pairs. We then calculated the proportion of these transitions that corresponded to the tested modification category as a function of the log2 expression difference. If this proportion for the experimental log-fold value exceeded the pre-established threshold q=0.7, the gene was classified as exhibiting the tested modification.

We show in Supplementary Figure 1 the probability distribution for the SNX18P8 gene as an example. For a given log-fold value, the figure shows the probability to discover a gene activation. Meaningless values above 1 are due to the interpolation function.

In Supplementary Figure 2 we compare the log-fold values corresponding to a probability q = 0.7 with the mean log-fold differential expression of genes. The latter was the criterion used in Ref. [13] to analyze the example on POM121 knockdown. The diagonal is drawn in red. The first quadrant corresponds to tumor-above genes, for which activation requires a positive log-fold value. And the third quadrant - to tumor-below genes. Note that the differential expression values largely underestimates the 0.7 threshold for activation. Thus, its use to discover activation/deactivation events introduces a systematic overcounting factor.

Experimental perturbation data

As mentioned in section 2.5 of Methods, we reanalyze the example treated in [13], and add two new examples involving NT genes: knockdown of the FGFRL1 gene in the PC3M cell line [27], and

knockdown/overexpression of the YAP1 gene in three prostatic cancer cell lines with the purpose of inducing/reversing the neuroendocrine phenotype [28].

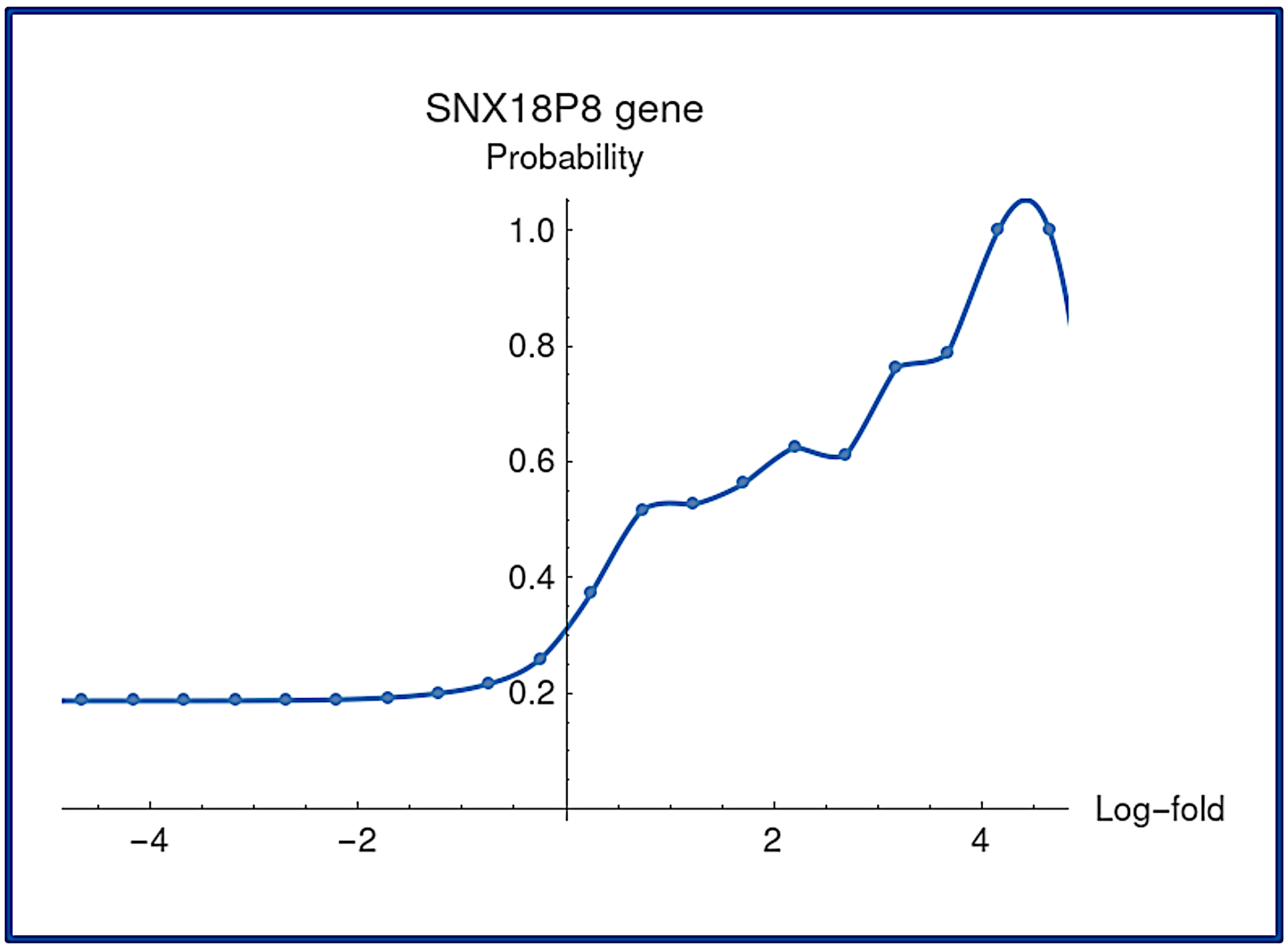

Supplementary Figure 1. Probability distribution for activation of the SNX18P8 gene.

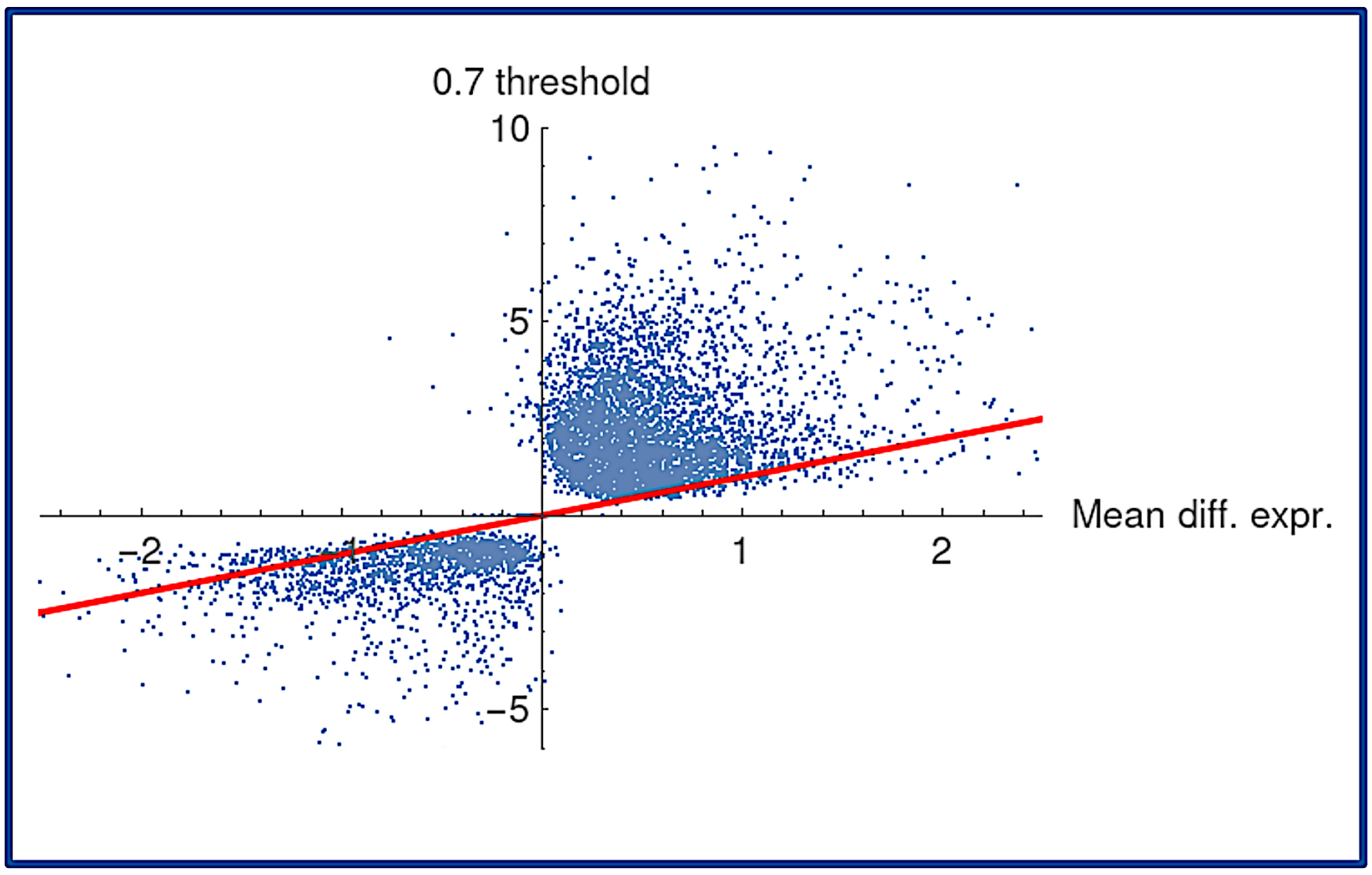

Supplementary Figure 2. Comparison between the mean differential expression of T-genes and the log-fold value corresponding to a probability equal to 0.7 for activation. TCGA data is used. The diagonal of the figure is marked in red.

Results

The results of the analysis are summarized in Supplementary Table I.

Target/ Cell line	POM121/ 22Rv1	POM121/ DU145	FGFRL1/ PC3M	YAP1/ H660	YAP1/ LNCap	YAP1/ DU145
Exp. type	KD	KD	KD	OE	KD	KD
Target type	Tumor-above	Tumor-above	Tumor-above/ normal-below	Normal-above/ tumor-below	Normal-above/ tumor-below	Normal-above/ tumor-below
Target Freq.	FrecT= 0.248	FrecT= 0.248	FrecT= 0.493 FrecN = 0.115	FrecT= 0.118 FrecN = 0.154	FrecT= 0.118 FrecN = 0.154	FrecT= 0.118 FrecN = 0.154
Target activ/deactiv Prob.	Prob(deactiv T) = 0.58	Prob(deactiv T) = 0.49	Prob(deactiv T) = 0.83 Prob(activ N) = 0.08	Prob(deactiv T) = 0.96 Prob(activ N) = 0.11	Prob(activ T) = 0.99	Prob(activ T) = 0.68
Total T-genes	4926 (3748/24/1154)	4738 (3599/24/1115)	4140 (4088/3/49)	2041 (203/33/1805)	2905 (271/50/2584)	2187 (187/27/1973)
Activated T-genes	10	10	40	1	40 (0/1/39)	7 (0/0/7)
Deactivated T-genes	41 (7/1/33)	22 (1/0/21)	85 (79/1/5)	5 (0/1/4)	30	6
Cascade genes/ Total Cascade	0/135 (reverse cascade)	0/133 (reverse cascade)	2/514 (reverse cascade, 4 steps)	0/46 (reverse cascade)	15/932 (direct cascade, 5 steps)	1/716 (direct cascade, 3 steps)
Quotes from the exps.	Measurements started 24 h after transfection? Gene knockdown efficiency was checked?		Failed experiments with VCap and LNCap cells? Essential gene?	Modest increase in neuroendocrine markers in the LNCap line, and still less effects in the DU145 line? Reduction in a single marker in the H660 line?		

Supplementary Table I. Summary of the experiments reported in [26-28]. Numbers in parenthesis, like (1/0/22), correspond to number of genes with frequency lower/equal/higher than the target frequency. KD – gene knockdown, OE – gene over expresion.

Trends:

- The first four experiments correspond to deactivation of a T-gene, The last two involve the activation of a T-gene. In case of FGFRL1 and YAP1, which are also NT-genes, N-activation is not achieved under the experimental conditions. Thus, we did not check activation of N-genes.

- The deactivation of POM121 does not reach the 0.7 threshold we require, in spite that authors claim that KD efficiency was checked.

- The first two, and probably all examined experiments, correspond to single-time perturbation with right after perturbation measurements. In a way, they report transient results, which we are not able to describe.

- In deactivation experiments, we mainly discovered deactivation events. In activation experiments – activation events.

- Spontaneous deactivation of genes should perhaps be included also in our scheme as a response to the intervention.

- Deactivated genes in the reverse cascade are rarely discovered. This is probably related to weak target deactivation or low target frequency.

- We verified that FGFRL1 is not an essential gene for viability in the Vcap and LNCap lines in the sense of CRISP screens [43]. However, it is a relatively high-frequency T-gene and may be related to an important property of the tumor phenotype.

In summary, the analyzed experiments neither validate nor disprove our theoretical scheme. In general, there are facts consistent with our predictions, and others for which we may find plausible explanations. Probably, specially designed experiments are needed.

Supplementary references

[43] Pacini C, Dempster JM, Boyle I et al. Integrated cross-study datasets of genetic dependencies in cancer. Nat Commun 2021;12:1661. doi:10.1038/s41467-021-21898-7
